# Intimate partner violence among women with eating disorders during the perinatal period

**DOI:** 10.1002/eat.22429

**Published:** 2015-05-29

**Authors:** Radha Kothari, Abigail Easter, Rebecca Lewis, Louise M. Howard, Nadia Micali

**Affiliations:** ^1^ Behavioural and Brain Sciences Unit, UCL Institute of Child HealthUCL Division of Psychology and Language SciencesLondonUnited Kingdom; ^2^ Section for Women's Mental HealthKCL Institute of PsychiatryLondonUnited Kingdom

**Keywords:** intimate partner violence, eating disorder, ALSPAC, prevalence, perinatal, pregnancy, physical IPV, emotional IPV, anorexia nervosa, bulimia nervosa

## Abstract

**Objective:**

Prevalence of intimate partner violence (IPV) during pregnancy is estimated to be 4%–8%. Women with mental health difficulties are at increased risk for IPV during the perinatal period. Prevalence of IPV is high among women with eating disorders (ED); however, prevalence of IPV during the perinatal period among women with ED is unknown.

**Method:**

We studied women from a population‐based cohort, the Avon Longitudinal Study of Parents and Children. Prevalence and odds of physical and emotional IPV during and after the perinatal period was investigated among women with lifetime ED, with (*n* = 174) or without pregnancy shape and weight concern and/or purging behaviors (*n* = 189), and women with no ED (*n* = 8723).

**Results:**

Women with lifetime ED showed higher prevalence of IPV during and after the perinatal period (physical = 9.6%–14.3% and emotional = 24.1%–28.1%). Lifetime ED was associated with higher odds of physical IPV during the perinatal period (odds ratio: 2.34, 95% confidence interval: 1.11–4.93, *p* = .03). Lifetime ED with and without pregnancy shape and weight concerns and/or purging was associated with higher odds of IPV after the perinatal period, and higher odds of reporting emotional IPV at all time points. Associations were moderated by partner's response to pregnancy and maternal experience of childhood sexual abuse.

**Discussion:**

Mothers with ED and their children may be vulnerable to negative effects due to maternal ED and IPV combined, both of which have been associated with severe and long‐lasting harmful consequences. Partner's response to pregnancy and maternal experience of childhood sexual abuse might contribute to the association between ED and IPV perinatally. © 2015 The Authors. International Journal of Eating Disorders published by Wiley Periodicals, Inc. (Int J Eat Disord 2015; 48:727–735)

## Introduction

Intimate partner violence (IPV), defined as acts of physical, sexual, or psychological abuse executed by a current or former partner, remains a serious and preventable public health issue and a substantial economic burden.^1^, ^2^ IPV has repeatedly been associated with significant physical morbidity and negative psychological consequences.^3^, ^4^ Prevalence of physical IPV during pregnancy has been estimated to be between 4% and 8%.^5^ This is of particular concern because of the negative psychological and health consequences to mother and child (including maternal and neonatal mortality), associated with experience of IPV during the perinatal period.^6^, ^7^, ^8^, ^9^ A recent review found that odds of experiencing IPV during the perinatal period were much higher among women with symptoms of depression, anxiety, and post‐traumatic stress disorder (PTSD) in comparison with women with no psychiatric history.^10^ This review highlighted a lack of research investigating prevalence and outcomes of IPV among women with eating disorders (ED) during the perinatal period, despite ED having been associated with a high prevalence of IPV over the lifetime,^11^ similarly to other psychiatric disorders.^12^

This gap in the literature needs to be addressed because the perinatal period has already been identified as a time of increased risk of negative consequences for women with ED and their children. Studies show that past and/or active ED are associated with adverse obstetric outcomes,^13^, ^14^, ^15^ infant feeding difficulties,^16^ and higher levels of psychopathology during and after pregnancy.^17^ Additional exposure to IPV during this period of increased risk may lead to even more negative consequences for women with ED and their children. Furthermore, pregnancy may be the ideal time to identify IPV among women with ED because of the high likelihood of women accessing healthcare during this period.^18^

This is the first study to investigate the prevalence and odds of IPV, during and after the perinatal period, among women who have had a lifetime ED (an ED over their lifetime, prior to or during pregnancy), and among women who additionally report presence of shape and weight concerns and/or purging during pregnancy, in comparison with women with no history of ED. Previous research investigating IPV among women with ED at other life points (not during the perinatal period) has been criticized for inadequately controlling for other factors that may influence the association between ED and IPV, particularly history of sexual abuse.^11^ Childhood sexual abuse has been associated with both ED^19^, ^20^, ^21^ and IPV in adulthood (revictimization).^22^, ^23^ It is possible that the relationship between ED and IPV during and after the perinatal period may be partly due to the high levels of childhood sexual abuse among women with ED,^11^ or other factors associated with the pregnancy itself. Because of this, a secondary aim of this research was to consider other factors that may influence the relationship between ED and IPV, specifically, (i) maternal experience of childhood sexual abuse and (ii) partner reaction to the pregnancy.

We hypothesized that lifetime ED would be associated with higher odds of experiencing physical and emotional IPV during the perinatal period in comparison with no history of ED. Although we explored whether additionally reporting marked shape and weight concerns and/or purging in pregnancy (as a proxy for active symptoms in pregnancy) would show differential associations with IPV, the insufficient evidence‐base prevented a directional hypothesis. With regard to our secondary aim, we hypothesized that maternal history of childhood sexual abuse and partner reaction to the pregnancy might contribute to the association between ED and IPV.

## Method

### Participants

The Avon Longitudinal Study of Parents and Children (ALSPAC) is a population‐based cohort of women and children. Pregnant women were eligible for recruitment if they lived in a predefined area of Bristol previously known as Avon, and if their expected date of delivery was between April 1, 1991 and December 31, 1992. Women enrolled in the study (*n* = 13,761) and their children (*n* = 13,867) have been followed up through a variety of questionnaires and clinics for the last 19–22 years.^24^

### Measures

#### Predictors

##### Maternal lifetime ED

At 12 weeks gestation, women were sent a questionnaire that included a brief self‐report screening for history of psychiatric illness.^13^ To determine whether mothers had experienced an ED at any point over their lifetime, they were asked whether they had ever had anorexia nervosa (AN) and/or bulimia nervosa (BN). Data on maternal history of ED were available for 12,254 women, of which 171 (1.4%) women reported history of AN, 199 (1.6%) reported history of BN, and 82 (0.7%) reported history of both AN and BN (AN + BN). This type of self‐report indicator of ED diagnosis has been validated in a population sample of pregnant women, with self‐reported lifetime AN showing a sensitivity of 100% and a specificity of 96%, and self‐reported lifetime BN showing a sensitivity of 94% and a specificity of 81%.^15^ Prevalence of ED in this sample is consistent with lifetime prevalence of ED in the general population,^25^ and research shows that self‐report measures such as those used in this study are just as good a screener as more commonly used measures.^26^

##### Pregnancy shape and weight concerns and/or purging

At 18 weeks gestation women were asked to answer questions from the Eating Disorders Examination Questionnaire^27^ regarding eating and weight concerns over the previous 28 days. Five of the nine items from the Shape Concern subscale, and five of six items from the Weight Concern subscale were included. It has previously been established that scores from these reduced subscales correlate significantly with the full subscales among women who have already been identified as having eating problems (weight concerns = 0.99 and shape concerns = 0.98).^28^ Furthermore, a previously defined mean score of ≥2 on either of these subscales has previously been used to determine whether women (previously identified as having eating problems) showed “marked shape or weight concern during pregnancy.”^28^ Women were also asked whether they employed purging behaviors for weight loss during the pregnancy under study (i.e., self‐induced vomiting and/or use of laxatives).^13^ For this study, women were defined as having *marked shape and weight concerns and/or purging* (coded as a binary variable: yes or no) if they had a mean score of ≥2 on the Eating Disorders Examination Questionnaire Weight Concerns or Shape Concerns subscales (the previously defined and validated cut‐off),^28^ and/or reported purging in pregnancy. Women reporting lifetime ED at 12 weeks gestation were divided into two groups based upon whether they also reported marked shape and weight concerns and/or purging at 18 weeks gestation (as a proxy for active concerns/symptomatology in pregnancy), creating a predictor variable with three levels: (i) no history of ED (*n* = 8,723, 96.0%); (ii) lifetime ED (*n* = 189, 2.1%); and (iii) lifetime ED with pregnancy shape and weight concerns and/or purging (*n* = 174, 1.9%).

#### Moderators/Mediators

##### Childhood sexual abuse

After a review of the literature and in collaboration with experts, seven questions were derived by ALSPAC regarding early sexual experience and were included in postal questionnaires at 32 weeks gestation after extensive piloting. Participants were asked whether the experience in question had occurred prior to 16 years of age (and how old they were), how many times it had happened, and whether it was desired. A question asking whether participants had been sexually abused before 17 years old was also included. This information was summarized to indicate whether mothers had experienced childhood sexual abuse; a binary variable was derived.

##### Partner's reaction to pregnancy

As part of the questionnaire sent out at 18 weeks gestation, mothers were asked how their partners had responded to the pregnancy with a choice of six possible responses: (i) overjoyed, (ii) pleased, (iii) mixed feelings, (iv) not happy, (v) very unhappy, and (vi) indifferent. A binary variable was derived for use in statistical analyses indicating whether the partner was happy (overjoyed or pleased) or not completely happy (mixed feelings, not happy, very unhappy, and indifferent).

#### Outcomes

##### Reports of physical and emotional IPV

A series of five self‐report questionnaires, designed and extensively piloted by ALSPAC, were sent to mothers over the perinatal period and included questions regarding their experience of IPV. Mothers were asked whether their partner had hurt them physically or been emotionally cruel, with a choice of five possible responses from a Likert scale. These responses ranged from “yes, and affected me a lot” or “yes, and affected me moderately” to “no, did not happen.” For the purpose of this study, responses were dichotomized to either yes (physical/emotional abuse experienced) or no (physical/emotional abuse not experienced). Each questionnaire asked these questions with reference to a specified period of time as follows: (1) at 18 weeks gestation, mothers were asked about the period since becoming pregnant; (2) at 8 weeks postnatal mothers were asked if abuse had been experienced since the middle of their pregnancy; (3) at 8 months postnatal they were asked about the period since the birth of their child; (4) at 21 months postnatal they were asked if abuse had been experienced since their child was 8 months old; and (5) at 33 months postnatal mothers were asked about the period since their child was 18 months old. Three outcomes were derived to represent the prenatal period (time points 1 and 2), the postnatal period (time point 3), and 8–33 months after delivery (time points 4 and 5). A timeline of all data collection can be found in Appendix 1.

#### Sociodemographic Data

Data on relationship status (married/cohabiting versus not), maternal education (up to and including O levels/GCSE equivalent versus A level and above), maternal ethnicity (white versus non‐white), and parity (primiparous versus multiparous) were collected via questionnaire during pregnancy, and data on maternal age were recorded at time of birth.

### Eligibility, Attrition, and the Sample under Study

Women were eligible for inclusion in prevalence estimates if data were available on self‐report of ED at 12 weeks gestation (*n* = 12,254). For 1,634 (13.3%) of the eligible sample, there were not enough data available on outcomes to determine the prevalence of IPV at any of the three time points under consideration (prenatal, postnatal, and 8–33 months after delivery), and these participants were excluded. Participants were less likely to have missing data if they were white (odds ratio (OR): 0.49; 95% confidence interval (CI): 0.31–0.78; *p* = .002), educated to at least secondary education standard (OR: 0.42; 95% CI: 0.34–0.53; *p* < .001), married/cohabiting (OR: 0.49; 95% CI: 0.41–0.57; *p* < .001), and older at the time of delivery (OR: 0.91; 95% CI: 0.90–0.93; *p* < .001). Sample sizes varied for each time point and are detailed in Table 1.

**Table 1:** Prevalence of physical and emotional IPV during and after the perinatal period among women with a lifetime eating disorder

		No ED		AN		BN		AN + BN	
	*N*	*n*	Prevalence	*n*	Prevalence	*n*	Prevalence	*n*	Prevalence
Prenatal period									
Physical IPV	9,969	8,298	282 (2.9%)	130	7 (5.0%)	157	6 (3.6%)	65	6 (8.6%)
Emotional IPV	10,041	8,904	962 (10.0%)	128	24 (17.5%)	160	34 (20.2%)	64	18 (26.1%)
Postnatal Period									
Physical IPV	10,620	8,186	218 (2.1%)	122	5 (3.5%)	153	8 (4.5%)	61	3 (3.8%)
Emotional IPV	10,620	8,186	811 (7.4%)	122	23 (16.2%)	153	25 (14.0%)	61	13 (18.3%)
8–33 months after delivery									
Physical IPV	8,680	6,833	433 (5.2%)	101	11 (9.6%)	126	14 (9.7%)	53	9 (14.3%)
Emotional IPV	8,812	6,912	1,431 (16.9%)	104	31 (26.3%)	127	35 (24.1%)	54	18 (28.1%)

Participants were eligible for inclusion if they had complete data on predictors, outcomes, partner reaction to pregnancy, and childhood sexual abuse. Sample sizes varied for each time point and are detailed in Table 2.

**Table 2:** Sample sizes for logistic regression analyses during the prenatal period, postnatal period, and 8–33 months after delivery

	No ED			ED (no concerns/symptoms)			ED (with concerns/symptoms)		
	No	Yes	Total	No	Yes	Total	No	Yes	Total
Prenatal									
Physical IPV	8,132 (98.0%)	166 (2.0%)	8,298	175 (95.6%)	8 (4.4%)	183	163 (96.4%)	6 (3.6%)	169
Emotional IPV	7,689 (92.6%)	615 (7.4%)	8,304	154 (84.6%)	28 (15.4%)	182	131 (77.1%)	39 (22.9%)	170
Postnatal									
Physical IPV	8,051 (98.4%)	135 (1.6%)	8,186	171 (96.6%)	6 (3.4%)	177	153 (96.2%)	6 (3.8%)	159
Emotional IPV	7,634 (93.3%)	552 (6.7%)	8,186	151 (85.3%)	26 (14.7%)	177	130 (81.8%)	29 (18.7%)	159
8–33 months after delivery									
Physical IPV	6,563 (96.0%)	270 (4.0%)	6,833	138 (90.8%)	14 (9.2%)	152	113 (88.3%)	15 (11.7%)	128
Emotional IPV	5,922 (85.7%)	990 (14.3%)	6,912	122 (79.2%)	32 (16.9%)	154	88 (67.2%)	43 (32.8%)	131
*Model 3: adjusted for partner reaction to pregnancy*									
Prenatal									
Physical IPV	8,065 (98.1%)	153 (1.9%)	8,218	170 (95.5%)	8 (4.5%)	178	105 (95.5%)	5 (4.5%)	110
Emotional IPV	7,634 (92.8%)	590 (7.2%)	8,224	149 (84.7%)	27 (15.3%)	176	83 (74.1%)	29 (25.9%)	112
Postnatal									
Physical IPV	7,966 (98.4%)	133 (1.6%)	8,099	166 (96.5%)	6 (3.5%)	172	99 (95.2%)	5 (4.8%)	104
Emotional IPV	7,551 (93.2%)	548 (6.8%)	8,099	147 (85.5%)	25 (14.5%)	172	84 (80.8%)	20 (19.2%)	104
8–33 months after delivery									
Physical IPV	6,514 (96.1%)	265 (3.9%)	6,779	134 (91.2%)	13 (8.8%)	147	68 (86.1%)	11 (13.3%)	79
Emotional IPV	588 (85.7%)	978 (14.3%)	6,859	119 (79.3%)	31 (20.7%)	150	48 (58.5%)	34 (41.5%)	82
*Model 4: adjusted for maternal childhood sexual abuse*									
Prenatal									
Physical IPV	7,382 (98.0%)	150 (2.0%)	7,532	165 (95.9%)	7 (4.1%)	172	155 (96.3%)	6 (3.7%)	161
Emotional IPV	6,965 (82.5%)	565 (7.5%)	7,530	146 (84.4%)	27 (15.6%)	173	125 (77.2%)	37 (22.8%)	162
Postnatal									
Physical IPV	7,311 (98.3%)	127 (1.7%)	7,438	161 (96.4%)	6 (3.6%)	167	145 (96.0%)	6 (4.0%)	151
Emotional IPV	6,930 (93.2%)	508 (6.8%)	7,438	142 (85.0%)	25 (15.0%)	167	123 (81.5%)	28 (18.5%)	151
8–33 months after delivery									
Physical IPV	6,022 (96.1%)	244 (3.9%)	6,266	130 (90.3%)	14 (9.7%)	144	107 (87.7%)	15 (12.3%)	122
Emotional IPV	5,416 (85.5%)	922 (14.5%)	6,338	118 (80.8%)	28 (19.2%)	146	83 (66.9%)	41 (33.1%)	124

### Statistical Analyses

The numbers of women reporting both lifetime ED (AN, BN, or AN + BN) and experience of IPV were low; therefore, ED groups were combined to increase power. Multiple logistic regression analyses were conducted to investigate the association between predictors and outcomes (Model 1). Potential confounders based upon evidence in the literature^29^ (maternal ethnicity, age, education, and parity) and variables that predicted attrition (ethnicity, maternal education, relationship status, and maternal age) were included in a second model (Model 2). Partner's reaction to the pregnancy was additionally included in a third model (Model 3), and maternal experience of childhood sexual abuse was included a fourth model (Model 4). All analyses were conducted using SPSS version 21 (SPSS, Chicago. IL), and a two‐tailed significance level of *p* ≤ .05 was used. Significance levels were not adjusted for multiple comparisons because analyses applied to different times over the perinatal period.

### Missing Covariate Data

Multiple random imputation was used to deal with missing covariate data. All predictor and outcome variables were used as predictors in the imputation model, which was set for 10 imputations. Missing data were imputed for relationship stability (1.3% missing). All analyses were run on complete case and imputed data sets and a sensitivity analysis showed that differences were negligible. Only results based on multiple imputation are presented here because complete case analysis suffers from more chance variation, and random multiple imputation is thought to correct any bias.

## Results

### Sociodemographic Characteristics

The percentage of women from ethnic minorities was low, and maternal age at delivery was similar in all three groups (see **Table** 3). A higher percentage of women with lifetime ED were educated to A‐level standard or above. The proportion of women that were married or cohabiting was highest among women without lifetime ED and lowest among women with lifetime ED plus marked weight and shape concerns and/or purging during pregnancy.

**Table 3:** Comparison of sociodemographic factors across eating disorder (ED) and non‐ED groups

	No ED	Lifetime ED	Lifetime ED with pregnancy shape/weight concerns and/or purging
Ethnicity: non‐white (vs. white), *n* (%)	190 (2.2)	4 (2.1)	6 (3.4)
Maternal education: A level^b^ and above (vs. up to O level), *n* (%)	3,276 (37.6)	105 (55.6)	68 (39.1)
Parity^a^: multiparous (vs. primiparous), *n* (%)	4,806 (55.1)	98 (51.9)	90 (51.7)
Relationship status: married/cohabiting (vs. single/widowed/divorced), *n* (%)	6,949 (79.7)	134 (70.9)	111 (63.8)
Age of mother at delivery (years), mean (SD)	28.5 (4.7)	28.2 (4.7)	29.5 (4.9)

a Parity = presence of other children at time of pregnancy (multiparous) or not (primiparous).

b A level and above equivalent in the United States to College Board Advanced Placement Exams or SAT II.

### Prevalence of IPV

Prevalence of physical and emotional IPV was higher among women with lifetime ED than those without and was highest for all women after the perinatal period (8–33 months after delivery). In most cases, differences in the prevalence of physical IPV between ED groups were small. Prevalence of emotional IPV was higher than physical IPV for all groups at all time points (see **Table** 1).

### Physical IPV

In comparison with women without ED, women with lifetime ED showed higher odds of reporting physical IPV during the prenatal period (OR: 2.24, 95% CI: 1.09–4.62, *p* = .03) and at 8–33 months after delivery (OR: 2.47, 95% CI: 1.40–4.33, *p* = .002), but not during the postnatal period (see **Table** 4). Differences remained significant in the fully adjusted model. In contrast, women with lifetime ED and pregnancy weight and shape concerns and/or purging had higher odds of reporting IPV during the postnatal period (OR: 2.34, 95% CI: 1.02–5.38, *p* = .05) and at 8–33 months after delivery (OR: 3.23 95% CI: 1.86–5.61, *p* < .001); however, in the fully adjusted model, differences remained significant only at 8–33 months after delivery (OR: 2.64, 95% CI: 1.49–4.68, *p* = .001). When adjusting for partner's reaction to the pregnancy (Model 3), women with lifetime ED continued to show significantly higher odds of reporting physical IPV during the prenatal period (OR: 2.33, 95% CI:1.10–4.94, *p* = .03) and the post‐perinatal period (OR: 2.59, 95% CI:1.44–4.64, *p* = .001). Women with lifetime ED and pregnancy shape and concerns and/or purging had higher odds of reporting physical IPV during the post‐perinatal period (OR: 2.29, 95% CI: 1.24–4.22, *p* = .01). When adjusting for maternal childhood sexual abuse (Model 4), women with lifetime ED and women with lifetime ED and pregnancy shape and weight concerns and/or purging continued to show significantly higher odds of reporting physical IPV at 8–33 months after delivery (after birth of child; OR: 2.77, 95% CI:1.54–4.99; *p* = .001 and OR: 2.61, 95% CI:1.46–4.67, *p* = .001, respectively).

**Table 4:** Associations between maternal exposure (ED) and physical IPV; unadjusted and adjusted odds ratios, 95% confidence intervals, and *p*‐values

	Model 1: OR (95% CI), *p*‐Values	Model 2: OR (95% CI), *p*‐Values	Model 3: OR (95% CI), *p*‐Values	Model 4: OR (95% CI), *p*‐Values
Prenatal				
No ED	Ref.	Ref.	Ref.	Ref.
Lifetime ED	2.24 (1.09–4.62), .03	2.34 (1.11–4.93), .03	2.33 (1.10–4.94), .03	2.20 (0.99–0.001), .05
Lifetime ED with pregnancy shape and weight concern and/or purging	1.80 (0.79–4.13), .16	1.45 (0.62–3.37), .40	1.45 (0.62–3.39), .40	1.31 (0.56–3.09), .53
Postnatal				
No ED	Ref.	Ref.	Ref.	Ref.
Lifetime ED	2.09 (0.91–4.81), .08	2.14 (0.92–4.98), .08	2.05 (0.87–4.80), .10	2.30 (0.98–5.42), .06
Lifetime ED with pregnancy shape and weight concern and/or purging	2.34 (1.02–5.38), .05	1.88 (0.81–4.37), .15	1.82 (0.77–4.27), .17	1.76 (0.75–4.14), .20
Post‐perinatal				
No ED	Ref.	Ref.	Ref.	Ref.
Lifetime ED	2.47 (1.40–4.33), .002	2.56 (1.43–4.59), .002	2.59 (1.44–4.64), .001	2.77 (1.54–4.99), .001
Lifetime ED with pregnancy shape and weight concern and/or purging	3.23 (1.86–5.61), <.001	2.64 (1.49–4.68), .001	2.29 (1.24–4.22), .01	2.61 (1.46–4.67), .001

Model 1 = unadjusted model. Model 2 = adjusted for maternal education, relationship status, maternal ethnicity, and parity. Model 3 = adjusted for maternal education, relationship status, maternal ethnicity, parity, and partners reaction to pregnancy. Model 4 = adjusted for maternal education, relationship status, maternal ethnicity, parity, and maternal childhood sexual abuse. Prenatal = during pregnancy. Postnatal = birth to 8 months postnatal. Post‐perinatal = 8–33 months after delivery. Lifetime ED (no pregnancy weight and shape concern/symptoms) = self‐report of ED over the lifetime at 12 weeks gestation without high shape and weight concerns and/or compensatory behaviors. Lifetime ED (with pregnancy weight and shape concern/symptoms) = self‐report of ED over lifetime at 12 weeks gestation with marked shape and weight concerns and/or compensatory behaviors.

### Emotional IPV

In comparison with women without ED, women with lifetime ED showed higher odds of reporting emotional IPV during the prenatal (OR: 2.27, 95% CI: 1.51–3.43, *p* < .001) and postnatal periods (OR: 2.38 95% CI: 1.56–3.64, *p* < .001), and also at 8–33 months after delivery (OR: 1.57, 95% CI: 1.06–2.33, *p* < .02) (**Table** 5). Women with lifetime ED and pregnancy weight and shape concerns and/or purging in pregnancy showed high odds of reporting emotional IPV during the prenatal (OR: 3.72, 95% CI: 2.58–5.37, *p* < .001) and postnatal periods (OR: 3.09, 95% CI: 2.04–4.66, *p* < .001), and also at 8–33 months after delivery (OR: 2.92, 95% CI: 2.02–4.24, *p* < .001). All differences remained significant in the fully adjusted model, and all but one of these differences remained significant when adjusting for partner's reaction to the pregnancy (Model 3), and when adjusting for maternal childhood sexual abuse (Model 4); in Models 3 and 4, women with lifetime ED no longer showed higher odds of reporting emotional IPV at 8–33 months after delivery.

**Table 5:** Associations between maternal exposure (ED) and emotional IPV; unadjusted and adjusted odds ratios, 95% confidence intervals, and *p*‐values

	Model 1: OR (95% CI), *p*‐Values	Model 2: OR (95% CI), *p*‐Values	Model 3: OR (95% CI), *p*‐Values	Model 4: OR (95% CI), *p*‐Values
Prenatal				
No ED	Ref.	Ref.	Ref.	Ref.
Lifetime ED	2.27 (1.51–3.43), <.001	2.11 (1.38–3.24), .001	2.12 (1.36–3.29), .001	2.11 (1.36–3.26), .001
Lifetime ED with pregnancy shape and weight concern and/or purging	3.72 (2.58–5.37), <.001	3.06 (2.09–4.50), <.001	2.85 (1.89–4.29), <.001	2.81 (1.89–4.19), <.001
Postnatal				
No ED	Ref.	Ref.	Ref.	Ref.
Lifetime ED	2.38 (1.56–3.64), <.001	2.23 (1.44–3.46), <.001	2.17 (1.39–3.38), .001	2.25 (1.44–3.53), <.001
Lifetime ED with pregnancy shape and weight concern and/or purging	3.09 (2.04–4.66), <.001	2.56 (1.67–3.91), <.001	2.57 (1.66–3.96), <.001	2.25 (1.44–3.53), <.001
Post‐perinatal				
No ED	Ref.	Ref.	Ref.	Ref.
Lifetime ED	1.57 (1.06–2.33), <.02	1.52 (1.01–2.27), .05	1.50 (1.00–2.25), .05	1.31 (0.85–2.01), .22
Lifetime ED with pregnancy shape and weight concern and/or purging	2.92 (2.02–4.24), <.001	2.60 (1.78–3.81), <.001	2.38 (1.60–3.53), <.001	2.48 (1.67–3.67), <.001

Model 1 = unadjusted model. Model 2 = adjusted for maternal education, relationship status, maternal ethnicity, and parity. Model 3 = adjusted for maternal education, relationship status, maternal ethnicity, parity, and partners reaction to pregnancy. Model 4 = adjusted for maternal education, relationship status, maternal ethnicity, parity, and maternal childhood sexual abuse. Prenatal = during pregnancy. Postnatal = birth to 8 months postnatal. Post‐perinatal = 8–33 months after delivery. Lifetime ED (no pregnancy weight and shape concern/symptoms) = self‐report of ED over the lifetime at 12 weeks gestation without high shape and weight concerns and/or compensatory behaviors. Lifetime ED (with pregnancy weight and shape concern/symptoms) = self‐report of ED over lifetime at 12 weeks gestation with marked shape and weight concerns and/or compensatory behaviors.

## Discussion

In this population‐based sample, the prevalence of both physical and emotional IPV was higher among women with a lifetime ED than women without. These findings confirm our hypothesis and are also in line with previous research showing high prevalence of IPV among women with depression, anxiety, and PTSD during the perinatal period.^10^ Although high prevalence of IPV was observed among women with lifetime ED during the prenatal and postnatal periods, it is particularly interesting to note that the prevalence of both physical and emotional IPV were highest at 8–33 months after delivery for all women. It is possible that this finding is indicative of a more general pattern of IPV increasing after the perinatal period within this sample.

Further statistical analyses showed that in comparison with women with no ED, women with lifetime ED had higher odds of experiencing physical IPV prenatally and from 8–33 months after delivery, but not during the postnatal period. Women who also reported marked shape and weight concerns and/or purging during pregnancy also had comparatively higher odds of reporting physical IPV from 8–33 months after delivery, but not during pre‐ or postnatal periods. In contrast to findings regarding physical IPV, experiencing of emotional IPV was more consistent over the three time periods. Women with a lifetime ED had higher odds of reporting emotional IPV than women without, and odds were highest for those women who additionally reported marked shape and weight concerns and/or purging during pregnancy.

Contrary to our hypothesis, during the postnatal period, women with a lifetime ED were not at greater risk of experiencing physical IPV than women without a lifetime ED, whether they did or did not additionally report pregnancy shape and weight concerns and/or purging. This could reflect a lack of power in the test, as is suggested by the low prevalence at this time point and the trend toward a significant difference consistent with other time points. The lack of association could also be due to women experiencing increased anxiety over this period regarding the disclosure of such personal information and concern about the possibility that their children could be removed by social services.^6^ Alternatively, the presence of an infant, who is likely to be in very close proximity to the mother during the postnatal period, may be protective against physical IPV. The clinical implications of this could be significant in that screening for IPV among women with a lifetime ED during the postnatal period may not be reliable (either because of nondisclosure or because physical IPV may have temporarily ceased at this time), warranting further research in this area. Health professionals should also be aware that women vulnerable to/with experience of physical IPV should be followed‐up even if they do not report IPV during the perinatal period, as it is possible that they will still be at risk during their child's early years.

Partner response to the pregnancy and maternal childhood sexual abuse were found to partly explain the association between maternal lifetime ED and physical IPV during the postnatal period, but not prenatally or during the post‐perinatal period (8–33 months post childbirth). This is particularly interesting because it suggests that although lifetime ED is independently associated with IPV during pregnancy and during the child's early years, other factors may play a greater role during the postnatal period. Among women who additionally reported pregnancy shape and weight concerns and/or purging, lifetime ED was only found to be associated with physical IPV at 8–33 months after delivery.

Lifetime ED was associated with emotional IPV after accounting for partner response to pregnancy or maternal childhood sexual abuse both prenatally and postnatally, but not at 8–33 months post‐pregnancy, though the trend toward significance at this time point and the low prevalence suggest that this may be due to a lack of power. Lifetime ED with pregnancy shape and weight concerns and/or purging remained associated with emotional IPV at all time points. Evidence suggests that early sexual abuse is a risk factor for later IPV.^22^, ^23^ Our findings suggest that it may be a moderator of the association between ED and physical IPV, and this should be further investigated in future studies.

It is estimated that by 18 years of age, 25% of young people have been witness to some level of domestic abuse or violence.^30^ Of particular concern is that domestic abuse and child maltreatment very often co‐occur, meaning that in homes where women experience IPV, children are at high‐risk of experiencing maltreatment themselves.^31^, ^32^ Based upon this evidence, our finding that women with a lifetime ED are more likely to experience physical and emotional IPV than women without could indicate that the children of women with a lifetime ED are also at higher risk of experiencing abuse. A high prevalence of psychological problems and difficulties in social understanding have been observed among children of mothers with ED,^33^, ^34^, ^35^ and one of the potential risk mechanisms for this may be via exposure to physical or emotional IPV. Health care professionals should be aware of this for early identification and prevention purposes.

This is the first study to investigate the prevalence of IPV during and after the perinatal period, among women with lifetime ED compared with women with no ED, but findings must be considered in light of strengths and limitations. Our sample was not only large but also drawn from a population‐based cohort, which is important with regard to the generalizability of findings to the whole population. Other strengths of this study are: the inclusion of a representative control group, the consideration of emotional as well as physical IPV, the availability of data from several time points over antenatal and postnatal periods, and the availability of sociodemographic data to account for confounding. There was, however, some attrition due to a small percentage of mothers not completing questionnaires during pregnancy. Also, the questions used to identify IPV reflect practice at the time (early 1990s), and were not action‐based questions, which are now known to increase the likelihood of disclosure.^36^ Partner's response to the pregnancy was reported by women themselves; therefore, results including this variable might be due to shared method variance. A high prevalence of interpersonal trauma and PTSD has been observed among women with ED, and both depression and anxiety are known to be highly comorbid with an ED diagnosis, all of which may also increase vulnerability for IPV.^37^ Women with ED are known to suffer from high comorbidity; therefore, adjusting for this would result in unrepresentative findings applicable to only a subgroup of women who have experienced ED only, without comorbidity.

Research investigating the frequency and outcomes of screening and intervention for IPV is limited,^38^ and future research should investigate whether current methods of screening and intervention are effective for mothers with history of mental illness. This is particularly relevant to women with ED, given the low disclosure of ED to antenatal healthcare professionals. Given that experience of IPV during the perinatal period is associated with higher risk of maternal and infant mortality,^39^ it is of vital importance that effective early interventions are developed for this group of women who are at particularly high risk. Larger longitudinal and prospective studies are required to investigate the prevalence of IPV among groups with ED across the lifespan as well as perinatally, particularly given our finding that both physical and emotional IPV increased markedly after the postnatal period. Future research should also investigate the mechanisms by which individuals with ED are at higher risk of experiencing IPV, because an understanding of these mechanisms may help in the development of targeted interventions.

## Appendix 1 A: Timeline of Data Collection

**Table:**

Prenatal period			Postnatal period	8‐33 months after deilvery		
12 weeks gestation	18 weeks gestation	32 weeks gestation	8 weeks postnatal	8 months postnatal	21 months postnatal	33 months postnatal
Maternal report of ED at any point over the lifetime	Maternal report of weight and shape concerns and/or purging behaviours over the previous 28 days	Maternal report of childhood sexual abuse	Maternal report of IPV since middle of pregnancy	Maternal report of IPV since birth of child	Maternal report of IPV since child was 8 months old	Maternal report of IPV since child was 18 months old
	Partner response to pregnancy					
	Maternal report of IPV since pregnancy					

The authors are extremely grateful to all the families who took part in this study, the midwives for their help in recruiting them, and the whole ALSPAC team, which includes interviewers, computer and laboratory technicians, clerical workers, research scientists, volunteers, managers, receptionists, and nurses. This publication is the work of the authors, and Radha Kothari, Abigail Easter, Rebecca Lewis, Louise Howard, and Nadia Micali will serve as guarantors for the contents of this article. The views expressed in this publication are those of the author(s) and not necessarily those of the NHS, the National Institute for Health Research, or the Department of Health. All work was conducted at the Behavioural and Brain Sciences Unit, Institute of Child Health, UCL.
